# Made to Measure: The Ethics of Routine Measurement for Healthcare Improvement

**DOI:** 10.1007/s10728-020-00421-x

**Published:** 2020-12-20

**Authors:** Polly Mitchell, Alan Cribb, Vikki Entwistle

**Affiliations:** 1 School of Education, Communication & Society, King’s College London, London, UK; 2 Health Services Research Unit and School of Divinity, History and Philosophy, University of Aberdeen, Aberdeen, UK

**Keywords:** Ethics, Healthcare improvement, Measurement, Continuous improvement, Patient experience

## Abstract

This paper analyses the ethics of routine measurement for healthcare improvement. Routine measurement is an increasingly central part of healthcare system design and is taken to be necessary for successful healthcare improvement efforts. It is widely recognised that the effectiveness of routine measurement in bringing about improvement is limited—it often produces only modest effects or fails to generate anticipated improvements at all. We seek to show that these concerns do not exhaust the ethics of routine measurement. Even if routine measurement does lead to healthcare improvements, it has associated ethical costs which are not necessarily justified by its benefits. We argue that the practice of routine measurement changes the function of the healthcare system, resulting in an unintended and ethically significant transformation of the sector. It is difficult to determine whether such changes are justified or offset by the benefits of routine measurement because there may be no shared understanding of what is ‘good’ in healthcare by which to compare the benefits of routine measurement with the goods that are precluded by it. We counsel that the practice of routine measurement should proceed with caution and should be recognised to be an ethically significant choice, rather than an inevitability.

## Introduction

Measurement is central to healthcare improvement. The 2008 National Health Service (NHS) report *High Quality Care For All* explicitly states what is often tacitly assumed: “we can only be sure to improve what we can actually measure” [1]. Assessing quality through measurement is increasingly taken to involve not just periodic audits or inspections, but routine measurement. The holy grail of such routine measurement practices is ‘continuous improvement.’ Though continuous improvement has been promoted for three decades [2], the widespread uptake of digital tools has transformed it from a distant ambition into something that looks increasingly like a practical possibility.

This paper takes stock of the ethics of building routine measurement for improvement into healthcare systems. We begin, in Sect. 2, by outlining the value of measurement and quantification in healthcare improvement. Thereafter, our argument is in two parts. First, in Sect. 3, we raise some relatively familiar questions about the instrumental effectiveness of measurement in bringing about improvements, suggesting that all-too-frequently it does not live up to its promise. Then, in Sect. 4, we argue that even if routine measurement for improvement does reliably lead to improvements, the practice has associated ethical costs. We argue that the practice of routine measurement has morally significant, transformative effects on the healthcare system and on professional practice. We identify and explore three such transformations: (a) changes to the definition of a ‘good’ *healthcare system*; (b) changes to the definition of ‘good’ *healthcare*; and (c) changes to the nature of accountability for healthcare decision-making. Making measurement and improvement into central functions of the healthcare system, we argue, creates conditions where certain activities, objects and aims can be easily seen to be valuable, and where the value of other activities, objects and aims can be obscured.

In Sect. 5, we conclude. The rise of routine measurement is unlikely to be reversible. We do not seek to suggest that it should be reversed—and certainly not in a blanket sense. Nor do we suggest that measurement or routine measurement are in themselves problematic or unethical. Nonetheless, routine measurement should proceed with the understanding that, as a practice, it has costs that are not straightforwardly justified or outweighed by the benefits that it brings. Throughout, we illustrate our discussion using examples from the measurement of patient experience, though the conclusions that we draw should not be taken to be limited to these cases alone.

## Measurement and Improvement

Measurement and healthcare improvement are such close bedfellows that the latter is commonly thought impossible without the former. The Institute for Healthcare Improvement (IHI), for instance, identifies “[a] clear, measurable aim” and “[a] measurement framework in support of reaching the aim” as necessary components of all improvement efforts [3]. The NHS Patient Safety First Campaign plainly states: “To demonstrate if changes are really improvement, you need the ability to test changes and measure the impact successfully” [4]. An NHS guide to improving healthcare services, which uses the IHI ‘Model For Improvement,’ asks aspiring improvers “How will we know if a change is an improvement?” and answers: “Measure the baseline,” “Measure regularly during testing,” “Continue to measure after the improvement is implemented” [5]. Measure, measure, measure.

So measurement has ‘epistemic value,’ that is, it can give us *knowledge* about healthcare processes and outcomes. And this knowledge can be used to justify claims about how good or bad healthcare is. Without measurement improvement efforts are based on guesses, intuitions and judgements, rather than knowledge. But epistemic value is not enough to secure the link between measurement and improvement. For that, measurement must also have ‘teleologic value,’ that is, it must serve the purpose for which it is intended. In this case, it must serve the ultimate end of improvement. In order to have such teleologic value, measurement must ground changes to health services that make the services better in some respect. Both the epistemic and teleologic value of measurement are forms of instrumental value—they characterise measurement as valuable insofar as it generates or leads to some other valuable ends.^1^ In this section, we set out how measurement realises these values.

Measuring aspects of inputs, processes and outcomes enables us to judge the effectiveness of interventions, and sometimes to infer causal relationships between different elements of a system [7]. It does so by providing a systematic representation of the features of processes and objects that are relevant to their performing defined functions or fulfilling their ends. Measurement can be used to create something approximating a ‘whole picture’ representation, allowing recognition of problems that are difficult to identify via the partial knowledge of processes that is available from any one vantage point. Measurement for improvement takes both quantitative and qualitative forms. Quantitative measurement uses numbers to represent characteristics of healthcare systems, such as mortality rates, disease incidence, bed days, and waiting times. Qualitative measurement uses chiefly textual data, from written documents, interviews, and surveys, for example, to develop a systematic representation of more nebulous characteristics such as organisational strategy, safety culture and patient-centredness. Measurement, and particularly quantification, can provide a common language for describing outcomes and outputs with some precision. It enables comparisons to be made across time, and across different teams and organisations. These characteristics underpin the central place of measurement in accountability and performance management systems. They can signal openness and transparency, as well as supporting audit and providing justification for decision-making.

The place of measurement in healthcare is often cemented by financial incentives such as pay-for-performance schemes and value-based pricing, as well as contractually and legally mandated publication of data and participation in clinical audits. These financial and institutional decision-making tools require widespread measurement in order to evidence their evaluation of healthcare services. The development of such mechanisms over the past several decades has been underpinned by a shift to an improvement mindset, whereby health systems and services are assessed and valued on the basis of their measured outcomes [8]. While there are other factors at play which motivate and shape measurement-based financial and institutional structures—notably relating to the relationship between healthcare institutions and government, insurers and pharmaceutical companies—they are explained and justified with reference to cost efficiency, waste reduction, and clinical effectiveness [9].

Routine measurement, that is, measurement as a part of day-to-day practice, is increasingly taken to be necessary for meaningful improvement. Measurement for improvement can take the form of regular audit or inspection checks to ensure continued compliance with good practice standards, but such checks are typically cyclical, rather than continuous. The move towards more regular, routine or even continuous measurement marks a move away from seeking to improve services by rooting out poor performance, and towards ‘continuous improvement.’ Continuous improvement, a concept that comes out of Japanese industry, sees poor performance as typically being built into systems and processes, rather than owing to the intentional behaviour of particular individuals. It involves identifying inefficient and wasteful aspects of processes and implementing interventions in an attempt to streamline systems. Don Berwick describes the underpinning theory:

Real improvement in quality depends, according to the Theory of Continuous Improvement, on understanding and revising the production processes on the basis of data about the processes themselves. "Every process produces information on the basis of which the process can be improved," say these theorists [2].

Routine measurement is thus central to continuous improvement, because it is needed to capture the information that is produced by the system. Routine measurement and continuous improvement are increasingly stated as core aims of healthcare systems. The Department of Health and Human Services in the United States (US), for example, defines healthcare quality improvement as “systematic and continuous actions that lead to measurable improvement in health care services” [10]. The NHS Constitution includes a commitment to continuous improvement:

You have the right to expect NHS bodies to monitor, and make efforts to improve continuously, the quality of healthcare they commission or provide. This includes improvements to the safety, effectiveness and experience of services [11].

Routine, widespread measurement and the continuous improvement framework are also advocated because of their potential to overcome some recognised problems with using measurement for performance management. ‘Name and shame’ approaches to healthcare improvement can lead people to falsify or massage data to improve perceived performance, and to spend needless time and resources attempting to disprove incompetence [12]. Such effects are particularly liable to arise when measurement is used as the basis for financial and status-related incentives such as pay-for-performance and value-based commissioning schemes. While cyclical measurement focusses on pre-specified targets, routine measurement also uses data to find and explore patterns that can inform quality improvement. Continuous improvement explicitly eschews a blame culture in favour of a collaborative approach to understanding why a system produces the outcomes that it does, and what changes can be made to improve upon them, thus disincentivising gaming and the manipulation of data [2].

So measurement and routine measurement have the potential to generate knowledge about healthcare processes and systems, and to underpin improvement efforts—that is, to produce objects of substantial value. However, in practice measurement does not always succeed in generating this value. Moreover, despite its benefits, routine measurement for improvement has side effects of its own, which suggest limits to its value.

## The Instrumental Limitations of Measurement for Improvement

While measurement can, in theory, lead to improvement in healthcare services, in reality it regularly fails to have such teleologic value, or leads only to modest improvements [13]. Patient experience provides a good example of the instrumental limitations of measurement for improvement because there is widespread failure to transform the measurement of patient experience into improvements in future patient experience. While we focus on examples from the measurement of patient experience in this section, the findings that we discuss support wide-ranging evidence of similar instrumental limitations in healthcare improvement efforts more generally [14–25].

Patient experience measures typically seek to capture how patients feel about the aspects of their care that are tangible to them: the convenience of the services provided, the environment within which care takes place, the ways healthcare professionals interact with them. Collecting such data and using it as part of an assessment of healthcare quality represents a recognition that the patient perspective is necessary for understanding the quality of healthcare services; knowledge of clinical outcomes, financial balance sheets and best practice guidelines are not enough. Measuring patient experience and sharing data analysis is presented as instrumental to improving patient experiences of healthcare in the future [1, 26].

The measurement of patient experience is increasingly routine and widespread [27]. In the US, the Centers for Medicare & Medicaid Services use several different patient experience surveys nationwide to assess service quality, and sometimes to determine the payments made to providers [28]. The NHS runs a series of patient experience surveys, covering inpatient care, emergency medicine, general practice, mental health, and social care [29–32]. Multiple measures can be, and are, used alongside one another, to build up a rich picture of patient expectations, experience and satisfaction with healthcare services [33]. Qualitative measures that are more narrative, in-depth and personal can complement the generalisable, numerical outputs of widely administrated surveys. However, the more in-depth, service-specific surveys are far more time and resource intensive to administer and evaluate than generic surveys with numerical outputs, so they inevitably include fewer patients [34]. If the measurement of patient experience is to be routine both in terms of regularity and spread, then realistically it must largely take the form of standardised surveys.

Baldie et al. argue that there are four key assumptions behind the idea that measuring patient experience can help improve patient experience [35]^2^:

1. Practitioners believe there are valid ways of assessing the healthcare experiences of patients for use in feedback.
2. Feedback of information about patients’ experiences to service providers stimulates improvement efforts.
3. Improvement efforts lead to observable changes in practice aimed at enhancing patients’ experiences.
4. Observable changes in practice in response to patient feedback lead to improvements in future patients’ experiences of healthcare.

The failure of any one of these assumptions can limit the value of measuring patient experience. Various studies have shown that clinicians can find measures of patient experience to be valuable and instrumental to improvement, at least in principle [36–41]. One study reported that “[d]octors who rejected the idea of feedback were regarded as depressed, alcoholic, burned-out, or just ‘bad doctors’” [39]. However, the validity and reliability of certain measures of patient experience are also called into question by clinicians [36, 39–41]. Concerns are raised about representativeness, the relevance of survey questions to patients, survey methodology and design. Of course, such concerns may be appropriate—some measures of patient experience will be invalid. But staff were more likely to question the measures and data when these were critical of services than when they were complimentary [34, 36, 42], which suggests that concerns are based, at least in part, on something other than the validity of measures.

Measurement of patients’ experiences can also fail to stimulate improvement efforts. For example, doctors and healthcare managers tend to only base improvement endeavours on patient experiences when measures identify problems with the service that they have already noticed themselves [38]. Improvement efforts are likely to focus on areas that are easier to change—such as food, the built environment or booking systems—rather than more intractable areas—such as communication and information provision [43]. Sometimes improvement efforts are broadly prompted by attempts to measure patient experience but end up focussing on areas not actually covered in the data that has been collected [37]. This looks to weaken the extent to which measuring patient experience for improvement can be said to reflect a patient-centred approach. More generally it should be clear that even where measures have unquestioned epistemic value they do not inherently have the desired teleologic value. For measures to drive improvements in the specific domains that are being measured key sets of actors have to be both motivated to respond to them (including by designing suitably responsive systems) and effective in so doing.

When improvement efforts are implemented, the likelihood of them leading to observable changes in practice is slim, and the chance of them actually leading to improvements in future patients’ experiences of healthcare is even slimmer. Many studies of the impact of patient experience measures focus only on their *perceived* impact and value by doctors and other healthcare professionals, and it’s unclear how much this reveals about genuine impact for patients [44]. Those studies that do measure actual impact on patient reported experience report very limited and mixed improvement outcomes [35, 44]. One improvement collaborative, for example, provided large amounts of support for healthcare providers to run improvement projects, in the form of planning meetings, written guides, data analysis and views, discussion of strategies and presentation of results [45]. However, only half of the providers surveyed actually implemented an improvement intervention, and none reported statistically significant improvements as the result of their efforts. The improvements that were observed were with respect to modest interventions which involved no major change in clinician behaviour.

Sometimes measuring patient experience does lead to improvements in future patient-reported experience. But here too there are caveats. While measuring experience can help to improve very poorly rated services, it may be much less effective at improving services that are already average or good [41, 46]. Moreover, a ‘first-time effect’ of measuring patient experience can be seen in poorly performing hospitals, with improvements diminishing in subsequent rounds of measurement and reporting [41]. This suggests de facto limits to continuous improvement. Patient feedback is judged by staff to be more useful if measurement outcomes are detailed and specific to their organisation, for example feedback from surveys containing free-text or from in depth interviews, and less helpful if it is generic [27, 41]. This indicates that the more generic surveys, which allow for wide comparison and less-resource intensive data collection and evaluation, may be less effective with respect to healthcare improvement.

So what is the upshot of these limitations? Each of the four key assumptions identified by Baldie et al. can, and at least sometimes does, fail to hold. This may indicate that much of the measurement of patient experience is unjustified. Asking patients to give feedback which is then ignored may be unethical [27], but so too is asking patients to give feedback in the knowledge that—even when it is not ignored—will not be epistemically or teleologically valuable in the ways that are invoked to justify its collection. Furthermore, the limitations of the measurement of patient experience for healthcare improvement are not just the result of poor data quality or insufficient data collection. They are also a result of the way in which data are used. Improving the validity of patient experience measures and increasing the amount of information that is collected may help to solve some of the limitations identified here, but certainly not all of them.

Evidence of similar instrumental limitations in other areas of healthcare improvement, besides patient experience, suggests that the measurement of healthcare services does not consistently serve healthcare improvement purposes in the ways that it is assumed and claimed to do. More, and more accurate, measurement will not necessarily rectify this or otherwise increase its value, because the failures to transform measurements into improvements are not only a matter of the quality and quantity of data, but a matter of how these data are understood and used.

Our discussion up to this point has been limited to the instrumental value and effects of the measurement. We have considered the goods that are intended to be produced by routine measurement, and the obstacles to it generating those goods. The second part of our argument considers the non-instrumental effects of routine measurement, including the goods that are embodied in practices of routine measurement, and the ways in which these can reconstitute healthcare.

## The Ethical Costs of Routine Measurement

Suppose that there are valid ways of routinely measuring patient experience, clinical effectiveness, safety, and so on, which reliably lead to observable changes in practice and, moreover, that these changes generate improvements that can be observed in future measures. Routine measurement for improvement would, in such circumstances, not only be possible, but would also have the desired teleologic value. But at what cost would this have been attained and would that attainment be worth the cost? In the remainder of the paper we consider the ethical costs of routine measurement for improvement.^3^

While routine measurement may have teleologic value when it succeeds in producing the desired causal consequences of routine measurement for improvement, it nonetheless has substantial transformative ‘side-effects.’ The practice of routine measurement reconstitutes and reframes healthcare in ways that result in an unintended transformation of the sector. The transformative costs of measurement are distinct from its instrumental costs and benefits, and not straightforwardly commensurable with them. For, as we will argue, the practice of routine measurement changes the way that value is understood in healthcare. That is, it creates conditions where certain activities, objects and aims can be easily seen to be valuable, and where the value of other activities, objects and aims can be obscured. We characterise the transformative costs of measurement as non-instrumental effects. They are not straightforwardly effects which enable or impede the attainment of particular valuable ends; rather, they enable and impede particular ways of valuing, and particular ways of understanding what is of value and what is not. When we call these effects ‘costs’ we don’t necessarily mean something wholly negative like ‘downside’ or ‘disadvantage’. Rather, we intend to capture something about what might be lost when healthcare is measured in a routine and widespread way. Sometimes such losses may be justifiable, but sometimes they will be unjustifiably burdensome. Sometimes they may be reversible—it may be possible to regain the precluded goods and values by simply ceasing to practice routine measurement—but at other times this might be difficult, for example, if the practice engenders deep-seated changes in social and ethical norms, expectations and behaviours.

The transformative effects of routine measurement come about chiefly because routine measurement changes the *function* of the healthcare system. Implementing routine measurement for improvement makes measurement and improvement into central functions of the healthcare system, alongside, for example, the delivery of clinical and therapeutic care. Measurement is sometimes taken to be a wholly descriptive practice—a matter of looking at an object or practice and empirically estimating the magnitude of its properties and relations. The object of measurement exists, and has properties and relations with given magnitude and nature, separately from its measurement. In a healthcare improvement context, however, it is difficult to maintain such an understanding of measurement. While measurement is sometimes carried out by third parties that are external to the practice of healthcare, more often it is embedded in healthcare institutions and practices. Even when measurement for healthcare improvement takes the form of audit and inspection, third parties typically use data that have been collected within healthcare institutions, by healthcare practitioners. Sometimes the parts of institutions that perform this function are compartmentalised—a hospital may have an audit department, improvement officers, a director of quality. However, more often, and particularly in the case of routine measurement, measurement is built into the day-to-day work of all employees. So, in order to routinely measure its practices, the roles of healthcare professionals and the activities that healthcare comprises undergo fairly significant changes. The object of measurement—the healthcare system and its processes—becomes something which also, simultaneously, performs a measurement function.

Moreover, routine measurement for improvement does not just add measurement and improvement to a list of existing functions of healthcare, rather, it reframes the existing functions of healthcare. If the processes and outcomes of healthcare must be measured, in order to be improved, then those processes and outcomes themselves need to be understandable—and understood—in metrical terms. Patient safety provides a good example of this. It is widely taken to be a central function of healthcare that it avoids causing unintended or unexpected harm to patients. Succeeding in this means not just avoiding actual harm, but also avoiding putting patients at high risk of harm—healthcare which involves a lot of ‘near misses’ is not safe healthcare. Once routine measurement is added to the functions of healthcare, the nature of patient safety changes. For now the epistemic standards attached to harm and risk of harm—that is, the standards for determining when and how it is known that patients are being harmed and being put at risk—are determined by measurement practices. Patient safety also changes when improvement is seen as a function of healthcare. Recent developments in the field of patient safety have moved away from an emphasis on avoiding making mistakes, where safety is understood as the absence of accidents and incidents [48]. Instead, safety is increasingly seen in terms of optimising system performance to ensure that things go right as often as possible—a much more aspirational model, which focuses on incremental improvement and harm reduction, rather than the avoidance of specified harmful events.

In his work on audit, Powers argues that audit does not just passively measure what is going on in organisations, but actively constructs them in its image [49]. That is, it encourages, and even requires, institutions to be auditable. We are suggesting that continuous measurement for improvement has the potential to do something similar. The prioritisation of routine measurement and improvement activities enables and encourages this redescription and redirection of core healthcare concepts and activities. And in this way, measurement and improvement become a central part of what it means to deliver healthcare, not something that is done in addition to delivering healthcare. We identify three changes effected by changing the function of the healthcare system in this way: first, routine measurement changes the definition of a ‘good’ *healthcare system*. Secondly, it changes definition of ‘good’ *healthcare*. Finally, it changes the nature of accountability for healthcare decision-making.

### Routine Measurement and the ‘Good’ Healthcare System

The first transformative effect of routine measurement for improvement is that the existence of the practice changes what it is to be a ‘good’ healthcare system.

The function of a complex social object such as a healthcare system is normatively inflected. That is, in expressing the function of the system we don’t merely describe what the system does, but also say something about what it *ought* to do. This is because such objects don’t just happen to exist, in comparison, perhaps, to geological objects or, more controversially, biological objects. Rather they are intentionally brought into existence, and designed and structured in order to bring about given ends and to fulfil particular purposes.^4^ The upshot of this is that expressing the function of such a system expresses something about what it would mean for that system to do well or badly with respect to those ends and purposes. When measurement and improvement become central parts of the function of a healthcare system, then, there is a change to what it means for it to be a ‘good’ healthcare system. That is, it is good not only insofar as it delivers healthcare, but also insofar as it measures and improves upon the delivery of healthcare.

This change in the meaning of what it is to be a ‘good’ healthcare system has notable ethical costs, which are most clearly seen when the aim of routine measurement is *continuous improvement*. When a framework of measurement for continuous improvement is introduced, the reframing of the function of the measurement system in terms of measurement and improvement is radical, because it tends to make healthcare functions into *maximising* functions. The goal of continuous improvement provides a perpetual reason to think that the healthcare system is not ‘good enough,’ and that there is always more that could, and should, be done. The function of the healthcare system therefore becomes not just to produce some defined set of goods, but to maximise those goods.

As a moral maximising function, continuous improvement rules out other kinds of moral reasoning as justification for healthcare system design and decision-making. One example of this is ‘satisficing’ or ‘sufficiency’ functions, which see the provision of ‘good enough’ healthcare to everyone as more morally significant than maximising health benefits. In theory, the continuous improvement framework treats every equally weighted improvement as having equal moral status. For example, there is no reason for thinking that improvements which bring one service up from poorly performing to average are any more significant than those which move another service from being good to excellent. Moreover, a commitment to continuous improvement need not involve a concern with equal distribution of healthcare services across a population. Conversely, if good healthcare is thought to be best justified by a satisficing principle, there will come a point, somewhere above a threshold level designating a basic minimum, where delivering further improvements becomes morally negligible. So, for example, once patients report good experiences of care, it might not be a moral priority to continue to routinely measure patient feedback and strive for marginal gains, especially where there are problems or holes elsewhere in the system. On a satisficing account, measurement and improvement should be prioritised in some areas over others, not because such a strategy is likely to lead to the greatest possible improvements, but because it is likely to lead to improvements which help to provide at least ‘good enough’ universal health coverage.

Adding a satisficing function to continuous improvement would thus undermine the ‘continuous’ nature of improvement`. Satisficing and continuous improvement are incompatible frameworks for thinking about good healthcare provision. This indicates one cost of continuous improvement, at least when that idea is unqualified or unspecified—certain principles guiding healthcare system design, resource distribution and justification of decision-making are ruled out insofar as they are incompatible with a maximising approach. More broadly, the case of continuous improvement illustrates the tendency for improvement not only to become an extra health service function but to significantly reshape and reconstitute health system functions by embodying assumptions about what matters in core healthcare architectures.

### Routine Measurement and ‘Good’ Healthcare

In addition to changing what it means for a *healthcare system* to be good, routine measurement for improvement also changes what it means for *healthcare* to be good. This is the second transformative effect of routine measurement for improvement.

The aim of improvement is, on its own, an insufficient guide to what is morally significant. Improvements will, plausibly, be judged on their extent—a larger improvement is, all things considered, better than a smaller one. But improvements may also be judged on their type: decreasing the number of avoidable deaths or the infection rate may, for instance, be deemed more morally significant than improving patient reported experience of the built environment in healthcare institutions. However, there is nothing inherent in the aim of improvement which settles the relative significance of different types of improvement. So while building measurement for improvement into the function of a healthcare system determines something about what it would mean for it to be a ‘good’ healthcare system—that it should seek to improve its processes and outcomes via measurement—it does not specify the content of such improvement—what we should measure and which measurement outcomes should be treated as better and worse. Substantive content must be provided by further specification of goals, the comparative value of different goods and ends, and constraints on actions and decisions.

Despite lacking such substantive normative content, measurement for improvement does limit the meaning of ‘good’ healthcare to some extent. Namely, when measurement and improvement are part of the central function of a healthcare system, good healthcare is understood in terms of measurable and measured processes and outcomes. The quality of care, and improvements to it, is assessed by collecting and evaluating measurements of it. What is measured matters—at least with respect to improvement and delivering good healthcare—more than what is not, because it determines what can be counted as an improvement and what cannot [50]. But not everything can be measured. In part this is a matter of resource constraint—there are an indefinite number of potential metrics, and it is not possible to capture all of them. Moreover, some things are more difficult to measure than others, either because there are not clearly agreed conventions around their measurement, or because measurement is particularly resource intensive—for example, it may be very time-consuming or the measuring tools very expensive. But there also seem to be some entities for which measurement can only capture a relatively small part of their extent or value [51]. A broad normative concept like ‘patient experience’ is perhaps this kind of entity. We can identify many plausible, even valid measures of patient experience, but they will not add together to exhaustively define patient experience. This is because patient experience is complex, multifaceted and multiply-realisable: there are many possible ways of defining and understanding patient experience, and particular measures of patient experience don’t straightforwardly reveal something definitive about how patients feel about their care. Instead each one constitutes a different measurable construct of ‘patient experience,’ the employment of which reveals something about that construct [52]. Each measure might be fit for purpose in some contexts, but will not reveal all that there is to know about patient experience.

There are a number of concerns with judging performance and making decisions on the basis of measurable attributes alone. There is reason to worry that orienting healthcare practitioners’ work towards measurement by defining improvement in terms of what is measured helps to equate this partial picture with good practice per se. A system that defines good healthcare practice in terms of particular measured processes or outcomes cannot distinguish between practice that is in fact good, and practice that merely meets the required performance targets [51]. There are well-documented problems of data fabrication, manipulation and ‘gaming’ in relation to performance management [53, 54]. But, in addition, meeting performance targets can belie a number of less fraudulent but nonetheless problematic scenarios, for example, “hitting the target and missing the point” [51], where measurable targets are met, but practice is not in line with the goals or normative concepts which prompted the measure to be developed in the first place. A target culture may discourage and erode the use of good clinical judgement (informed by caring and respectful discussion with the patient) about the inappropriateness of particular processes or outcome goals in particular cases. And performance can easily remain or become poor in areas where it is not measured or not measurable, representing a lack of managerial control in some parts of the organisation under the veneer of total control via measurement [52]. These problems emerge in part because measurement captures only a partial picture of good practice, especially where complex normative concepts are involved.

So, for example, if good patient experience is a necessary part of good healthcare, then good healthcare will be healthcare which patients experience as good. But this does not mean that improving patients’ experience of healthcare necessarily improves healthcare: the fact that healthcare is experienced as good does not mean that it is good healthcare.^5^ There are ways of improving patients’ experiences of healthcare which do not in fact reflect good healthcare. If the measures of patient experience are taken to be the only means of assessing patient experience of healthcare, then improving patient experience healthcare will involve improvement along these measured attributes. But focussing on improving measures of patient experience may focus on the wrong thing, if not all healthcare that is experienced as good according to measures *is* in fact good in all important respects. In general, if measures are indicators or signs of good practice rather than identical with good practice, then improving the indicators need not entail improved practice.

Measurement for improvement, then, changes what it means for healthcare to be good by reconceptualising ‘good’ in metrical terms. This can preclude objects and ends that are less easily measured from being recognised as valuable, and systematically prioritise measured goods.

### Routine Measurement and Accountability

The third transformative effect of routine measurement is that it changes the nature of accountability for healthcare decision-making.

Measurement is often seen as necessary for accountability [55–57]. To be accountable is to be responsible for something and, moreover, to be expected to provide a reason or justification for it—explaining why it is valuable or necessary, for instance. Measurement is taken to play an important role in explaining and justifying actions. Measuring aspects of inputs, processes and outcomes can provide evidence of the value of actions and interventions, as well as the value of organisations. Measurement can help third parties to understand the grounds for particular decisions. It is down to the features of measurement discussed above in Sect. 2, such as its systematicity and comparability, that measurement plays this role in accountability. Measurements, in theory, provide evidence that is less partial than the opinions and judgements of individuals, and which can be compared across different institutions, individuals and across time.

This move towards a measurement-centred conception of accountability changes aspects of relationships in healthcare. Concerns in this area are sometimes summarised as about a deterioration of trust between the public and healthcare institutions. Whilst we do not accept all the framings of such concerns about trust, we do think certain uses of routine measurement have the potential to corrode relationships and trust. Grounding accountability in measurement might be thought to lead to a deterioration in trust between the public and healthcare institutions, or between particular patients and healthcare staff. In a classic essay, Tsoukas argues that: “the more information on the inner workings of an expert system observers seek to have, the less they will be inclined to trust its practitioners; the less practitioners are trusted, the less likely it is for the benefits of specialized expertise to be realized” [58]. In other words, accountability that is grounded in measurement seems to be based on *mistrust*—measurable evidence of actions and their consequences are deemed acceptable justification, whereas personal judgements and assurances from hospital staff, are not. If patients are encouraged to carefully examine the performance of their doctors and nurses before agreeing to consultation or treatment, this suggests a lack of trust, both in the individuals involved, but also in the education and training system of which they are a product. However, there are limits to the extent to which measurement should be thought to undermine trust. Trust, on the whole, *is* subject to evidence. To trust a person or institution without any evidence for their trustworthiness, or to trust them despite evidence that they are not trustworthy, is likely a foolhardy strategy. And certain forms of checking and monitoring are compatible with, and even necessary for, trust [59, 60]. Perhaps the evidence provided by routine measurement for performance management and improvement is just the kind of evidence that is necessary for patients to trust clinical staff and institutions. If so, the fact that routine measurement is used as evidence for assessments of accountability need not in itself suggest a deterioration in trust. On the other hand, continuous checking and accounting may be incompatible with trust, which does seem to require granting *some* discretion to the person who is trusted [61]. So there may be certain uses of routine measurement for improvement which do lead to or represent a deterioration in trust.

Regardless, treating measurement as necessary for accountability does shape the way in which healthcare can be valued. As discussed above, measurement generates a partial picture of practice. It picks out certain things as markers of good and bad care, and—by omission— treats other things as less relevant. Taking measurement to be necessary for accountability, and a primary means of assuring accountability, fixes those things that are markers of good and bad care—that is, the things that are measured—and the kind of reasoning and justification that can be accepted as part of assurance and assessment of accountability—justification that refers to changes in measurement outcomes. Those processes and outcomes that are less easily measured will be less likely to be judged to be valuable. This is likely to impact some healthcare sectors and practices more than others, particularly those with more ‘soft’ and ‘indirect’ outcomes, such as social care, mental health and public health.^6^

The measurement of patient experience again provides a good example of the way in which the role of measurement in accountability shapes what can be counted as good healthcare. The questions in surveys of patient experience send clear signals about what the survey designers and distributors think patients should expect to receive from healthcare staff. Patients are questioned about particular attributes of their healthcare experiences regardless of how important these are to them. The best measures will be validated to ensure that they in fact measure what they purport to measure. But, as noted above, this cannot ensure that they capture all things that impact on patient experience. Assessment of care as good, with respect to patient experience, depends on patients answering these questions positively, or giving high scores. So doctors perform well, with respect to patient experience, if they can be shown to successfully deliver on this set of measurable ends—spending a specified amount of time with each patient, for example, offering an appointment within a set time, or not leaving patients waiting a long time for treatment. Without evidence from patient experience measures, it can be difficult to prove that something matters to patients or contributes to good or poor experiences of clinical care, if measures are taken to be central to understanding how the system operates. So those characteristics of care that are captured in patient experience surveys become the public face of patient centredness and patient experience, and the characteristics on the basis of which an institution or individual can be praised or criticised.

Building measurement into accountability shapes and limits the kinds of justification that can be given for healthcare decisions and practices. This is liable to determine the kinds of practices that are deemed acceptable or successful, favouring practices that maximise particular kinds of health benefit for populations, and which are amenable to routine measurement, even when such practices may be inappropriate or less good for some people. Such changes in the terms in which justification can be provided might also be taken to limit the scope for trust in the healthcare system. While trust in healthcare providers could be more readily justified with respect to the standards of what’s measured—because appropriate evidence for trusting attitudes can be provided in these areas—this is not the case with respect to non-measurable aspects of healthcare.

## Routine Measurement: is it Worth it?

We have argued that routine measurement for healthcare improvement changes the healthcare system in ethically significant ways. It changes the function of the system, and because of this it changes what it is to be a good *healthcare system*, what it is to be good *healthcare*, and how providers and staff can be held to account for the quality of services. But do the professed benefits of routine measurement and improvement make up for the things of value which they preclude?

Although this question is rarely posed so explicitly, in practice there seems to be a widespread presumption of a positive answer to it. However, it is a difficult question to answer because there is no single overarching framework available by which to compare a healthcare system transformed by routine measurement for improvement with one not so transformed. The conception of ‘good healthcare’ as constructed by routine measurement is inextricable from the value of measurement and data collection. There may be no shared understanding of what is good in healthcare by which to compare the goods of routine measurement with the goods that are precluded by it. In this sense the losses engendered by a move to routine measurement might be ‘tragic’ in the sense elucidated by MacIntyre [66], that is, it is possible that they cannot be fully, or perhaps even partially, compensated for by the goods which seek to replace them. Furthermore, once a new measurement-constrained value framework becomes embedded and normalised, it may become increasingly difficult even to recognise the good in displaced frameworks. Embedding routine measurement and continuous improvement in the healthcare system has the potential to change the ethical landscape of healthcare, by shaping and limiting what kind of activities, objects and ends are seen as valuable, and the ways that they can be valued.

Despite offering a sceptical discussion of routine measurement for improvement, and more specifically some of the limitations of the idea of continuous improvement, we do recognise the value and social power of the ‘measure and improve’ approach to healthcare, particularly with respect to very poor practice. Furthermore, routine measurement, and the conception of improvement it produces, is unlikely to go away any time soon. However, the arguments we have rehearsed here indicate the need to reflect on *how* routine measurement is taken forward.

Overall we argue that the further spread of routine measurement needs to be accompanied by a degree of routine scepticism and that this may entail adjustments both to the pace at which it is implemented, the kinds of areas where it is applied and the ways it is used. The practice of routine measurement for improvement is a choice and not an inevitability. The notion of continuous improvement in particular is just one way of conceptualising the function, role and trajectory of a healthcare system. Furthermore, routine measurement for improvement is a *normative* choice*;* a choice that reflects and establishes a particular conception of the good—and in the case of continuous improvement specifically represents a maximising framework for thinking about the good in healthcare. Such a normative framework may be more appropriate for some parts of the healthcare system than others, for example, there may sometimes be reason to prioritise other values over continuous improvement. It is a choice which precludes, but does not necessarily offset, other values and conceptions of the good in healthcare. A continuous improvement framework may not, for instance, be appropriate where there are areas of healthcare delivery that are clearly below a reasonable minimum—a satisficing approach might make better sense of improvement priorities. Moreover, routine measurement should be understood as one source of information about how good or bad healthcare services are, but not exhaustive, nor able to supplant non-routine means of coming to understand healthcare processes, such as inspections, ethnographies and the experiences of healthcare practitioners. Publication of data about healthcare organisations, and rankings of organisations, should proceed with caution, and not with a ‘more is better’ attitude.

In the first part of our argument we suggested that, and showed how, the measurement of patient experience fails to deliver on its promise. We do not wish to over-generalise from this one cluster of examples, but these findings support evidence that routine measurement for improvement is often ineffective on its own terms in other areas of healthcare; that is, it may not transform healthcare in intended ways. In the second part of our argument we have added in more fundamental concerns about the very significant unintended transformations that may come about as a result of routine measurement for improvement. The potential transformations in healthcare’s functions, professional roles and forms of accountability are both practically and morally substantial. We recognise that dominant structures and expectations within healthcare systems mean that individual actors may often have comparatively little elbow room in this regard and, of course, that—even if, and after, the implications of scepticism about measurement are accepted—routine measurement for improvement has a valuable contribution to make in many areas. Nonetheless, the use of routine measurement in healthcare does not represent an unequivocal good. We suggest that there are therefore good reasons for individual actors who are planning specific health services with improvement in mind to be cautious both about the emphasis they give to embedding routine measurement in their plans, and the ways in which they apply and interpret such measurement.

## Footnotes

1 In this paper we invoke the concepts of instrumental value and non-instrumental value. Instrumental value is the value something has in virtue of being a means to an end. Non-instrumental value (or final value) is the value something has as an end in itself. We broadly follow Christine Korsgaard in taking this distinction to be orthogonal to the distinction between intrinsic and extrinsic value, that is, between the value that something has ‘in itself’ or ‘for its own sake,’ and the value that something has in virtue of its relationship to other things [6]. We are not directly concerned with intrinsic and extrinsic value here, and our discussion instead focuses on instrumental and non-instrumental value.

2 Baldie et al. focus on general practice, but their programme theory applies to measures of patient experience and improvement efforts more generally.

3 There are, of course, financial costs to routine measurement, including routine measurement of patient experience [47]. It is, in theory, possible to calculate the financial cost of routine measurement and this figure could be included in an evaluation of the impact of such data collection with respect to healthcare improvement, though, in practice, such calculations are rarely made. Financial cost can be treated as roughly commensurable with the instrumental value of targeted improvements; improvements in patient experience, clinical effectiveness or timeliness, for example, can be said to be worth the additional financial cost of measurement and improvement initiatives (or not). Such trade-offs are complex and controversial, but they involve ethical reasoning of a sort that is well established in healthcare decision-making. The ethical costs of routine measurement that we identify in this section are different in an important way from the kinds of cost that can be included in such trade-offs. In what follows we focus on some of the non-instrumental costs of routine measurement, which have been relatively neglected in critiques and discussion of measurement in healthcare.

4 The same appears to be true of less complex objects that are the result of intentional design—we don’t want to say that the function of a kitchen knife is to injure people, even if that in fact describes how, on occasion, it is used.

5 Our argument here identifies an example of the logical fallacy ‘affirming the consequent.’ Compare the following two (invalid) syllogisms:

1. If this is good healthcare, then this will be experienced as good healthcare.
  This is experienced as good healthcare.
  Therefore, this is good healthcare.
2. If this is a cat, then this is a mammal.
  This is a mammal.
  Therefore, this is a cat.

6 Indeed, these are the areas where funding has been cut for health services in the UK over the past decade [62–65].

## References

[1] Darzi A. (2008). *High quality care for all: NHS Next Stage Review final report*. London, UK: UK Department of Health.

[2] Berwick DM (1989). Continuous improvement as an ideal in health care. New England Journal of Medicine.

[3] Institute for Healthcare Improvement (2019). Science of Improvement. Webpage. http://www.ihi.org/about/Pages/ScienceofImprovement.aspx. Accessed 3 Sep 2019.

[4] Clarke J, Davidge M, James L (2009). The how-to guide for measurement for improvement.

[5] NHS Improving Quality (2014). First steps towards quality improvement: A simple guide to improving services.

[6] Korsgaard K (1983). Two Distinctions in Goodness. The Philosophical Review.

[7] Donabedian A (1978). The quality of medical care. Science.

[8] Raftery J (2013). Value based pricing: can it work?. BMJ.

[9] Dixon J (2004). Payment by results—new financial flows in the NHS. BMJ.

[10] Department of Health and Human Services (2011). Quality improvement.

[11] NHS (2015). The NHS constitution.

[12] Pringle M, Wilson T, Grol R (2002). Measuring “goodness” in individuals and healthcare systems. BMJ.

[13] Dixon-Woods M, Martin GP (2016). Does quality improvement improve quality?. Future Hospital Journal.

[14] Anthony T, Murray BW, Sum-Ping JT, Lenkovsky F, Vornik VD, Parker BJ, McFarlin JE, Hartless K, Huerta S (2011). Evaluating an evidence-based bundle for preventing surgical site infection: a randomized trial. Archives of Surgery.

[15] Benning A, Ghaleb M, Suokas A, Dixon-Woods M, Dawson J, Barber N, Franklin BD, Girling A, Hemming K, Carmalt M (2011). Large scale organisational intervention to improve patient safety in four UK hospitals: Mixed method evaluation. BMJ.

[16] Benning A, Dixon-Woods M, Nwulu U, Ghaleb M, Dawson J, Barber N, Franklin BD, Girling A, Hemming K, Carmalt M (2011). Multiple component patient safety intervention in English hospitals: Controlled evaluation of second phase. BMJ.

[17] Coory M, White VM, Johnson KS, Hill DJ, Jefford M, Harrison S, Winship I, Millar J, Giles GG (2013). Systematic review of quality improvement interventions directed at cancer specialists. Journal of Clinical Oncology.

[18] Morello RT, Lowthian JA, Barker AL, McGinnes R, Dunt D, Brand C (2013). Strategies for improving patient safety culture in hospitals: A systematic review. BMJ Quality & Safety.

[19] Nadeem E, Olin SS, Hill LC, Hoagwood KE, Horrowitz SMC (2013). Understanding the components of quality improvement collaboratives: A systematic literature review. The Milbank Quarterly.

[20] Urbach DR, Govindarajan A, Saskin R, Wilton AS, Baxter NN (2014). Introduction of surgical safety checklists in Ontario Canada. New England Journal of Medicine.

[21] Etzioni DA, Wasif N, Dueck AC, Cima RR, Hohmann SF, Naessens JM, Mathur AK, Habermann EB (2015). Association of hospital participation in a surgical outcomes monitoring program with inpatient complications and mortality. JAMA.

[22] Mason SE, Nicolay CR, Darzi A (2015). The use of Lean and six sigma methodologies in surgery: A systematic review. The Surgeon.

[23] Barker AL, Morello RT, Wolfe R, Brand CA, Haines TP, Hill KD, Brauer SG, Botti M, Cumming RG, Livingston PM (2016). 6-PACK programme to decrease fall injuries in acute hospitals: Cluster randomised controlled trial. BMJ.

[24] Zegers M, Hesselink G, Geense W, Vincent C, Wollersheim H (2016). Evidence-based interventions to reduce adverse events in hospitals: A systematic review of systematic reviews. British Medical Journal Open.

[25] Zhong W, Feinstein JA, Patel NS, Dai D, Feudtner C (2016). Tall man lettering and potential prescription errors: a time series analysis of 42 children's hospitals in the USA over 9 years. BMJ Quality & Safety.

[26] Balik B, Conway J, Zipperer L, Watson J (2011). Achieving an Exceptional patient and family experience of inpatient hospital care. IHI innovation series white paper.

[27] Coulter A, Locock L, Ziebland S, Calabrese J (2014). Collecting data on patient experience is not enough: They must be used to improve care. BMJ.

[28] Centers for Medicare & Medicaid Services (2019). Consumer Assessment of Healthcare Providers & Systems (CAHPS). Webpage. https://www.cms.gov/Research-Statistics-Data-and-Systems/Research/CAHPS/. Accessed 3 Sep 2019.

[29] NHS Digital (2018). Social care user surveys. Webpage. https://digital.nhs.uk/data-and-information/data-collections-and-data-sets/data-collections/social-care-user-surveys. Accessed 3 Sep 2019.

[30] NHS Digital (2019). Friends and family test (FFT) data collection. Webpage. https://digital.nhs.uk/data-and-information/data-collections-and-data-sets/data-collections/friends-and-family-test-fft-data-collection. Accessed 3 Sep 2019.

[31] NHS Surveys (2019). Current surveys. Webpage. https://www.nhssurveys.org/surveys. Accessed 3 Sep 2019.

[32] GP Patient Survey. About the survey. Webpage. https://www.gp-patient.co.uk/about. Accessed 3 September 2019.

[33] de Silva, D. (2013). *Measuring patient experience*. Evidence Scan No. 18. London, UK: The Health Foundation.

[34] Ahmed F, Burt J, Roland M (2014). Measuring patient experience: Concepts and methods. The Patient.

[35] Baldie DJ, Guthrie B, Entwistle V, Kroll T (2017). Exploring the impact and use of patients’ feedback about their care experiences in general practice settings—a realist synthesis. Family Practice.

[36] Asprey A, Campbell JL, Newbould J, Cohn S, Carter M, Davey A, Roland M (2013). Challenges to the credibility of patient feedback in primary healthcare settings: a qualitative study. British Journal of General Practice.

[37] Barr J.K., Giannotti T.E., Sofaer S., Duquette C.E., Waters W.J., Petrillo, M.K. (2006). Using public reports of patient satisfaction for hospital quality improvement. *Health Services Research*, 41(3p1), 663–682. 10.1111/j.1475-6773.2006.00508.x PMC1713194 16704506

[38] Boiko O, Campbell JL, Elmore N, Davey AF, Roland M, Burt J (2015). The role of patient experience surveys in quality assurance and improvement: A focus group study in English general practice. Health Expectations.

[39] Edwards A, Evans R, White P, Elwyn G (2011). Experiencing patient-experience surveys: A qualitative study of the accounts of GPs. British Journal of General Practice.

[40] Heje HN, Vedsted P, Olesen F (2011). General practitioners' experience and benefits from patient evaluations. BMC Family Practice.

[41] Riiskjær E, Ammentorp J, Nielsen JF, Kofoed P-E (2010). Patient surveys—a key to organizational change?. Patient Education and Counseling.

[42] Davies E, Cleary PD (2005). Hearing the patient’s voice? Factors affecting the use of patient survey data in quality improvement. BMJ Quality & Safety.

[43] Draper M, Cohen P, Buchan H (2001). Seeking consumer views: What use are results of hospital patient satisfaction surveys?. International Journal for Quality in Health Care.

[44] Reinders ME, Ryan BL, Blankenstein AH, van der Horst HE, Stewart MA, van Marwijk HWJ (2011). The effect of patient feedback on physicians' consultation skills: A systematic review. Academic Medicine.

[45] Davies E, Shaller D, Edgman-Levitan S, Safran DG, Oftedahl G, Sakowski J, Cleary PD (2008). Evaluating the use of a modified CAHPS® survey to support improvements in patient-centred care: Lessons from a quality improvement collaborative. Health Expectations.

[46] Carey RG (2002). Improving patient satisfaction: A control chart case study. The Journal of Ambulatory Care Management.

[47] Loeb JM (2004). The current state of performance measurement in health care. International Journal for Quality in Health Care.

[48] Hollnagel, E., Wears, R.L. and Braithwaite, J. (2015). From safety-i to safety-II: A white paper. The Resilient Health Care Net: Published simultaneously by the University of Southern Denmark, University of Florida, USA, and Macquarie University, Australia.

[49] Powers M (1999). The audit society: Rituals of verification.

[50] Loxterkamp D (2013). Humanism in the time of metrics. BMJ.

[51] Bevan G, Hood C (2006). What’s measured is what matters: targets and gaming in the English public health care system. Public Administration.

[52] Pflueger D (2015). Accounting for quality: On the relationship between accounting and quality improvement in healthcare. BMC Health Services Research.

[53] Bird S, Cox D, Farewell VT, Goldstein H, Holt T, Smith PC (2005). Performance indicators: Good, bad, and ugly. Journal of the Royal Statistical Society.

[54] Mannion R, Braithwaite J (2011). Unintended consequences of performance measurement in healthcare: Twenty Salutary lessons from the NHS. Internal Medicine.

[55] Kazandjian VA (2002). Accountability Through Measurement: A Global Healthcare Imperative.

[56] Chassin MR, Loeb JM, Schmaltz SP, Wachter RM (2010). Accountability Measures—Using Measurement to Promote Quality Improvement. New England Journal of Medicine.

[57] Forster AJ, van Walraven C (2012). The use of quality indicators to promote accountability in health care: The good, the bad, and the ugly. Open Medicine.

[58] Tsoukas H (1997). The tyranny of light: The temptations and the paradoxes of the information society. Futures.

[59] O'Neill O (2002). A Question of Trust: The BBC Reith Lectures 2002.

[60] Entwistle VA, Quick O (2006). Trust in the context of patient safety problems. Journal of Health Organization and Management.

[61] Baier, A. (1991). Trust. The Tanner Lectures on Human Values. Princeton University, 6–8 March 1991. Resource document. https://tannerlectures.utah.edu/_documents/a-to-z/b/baier92.pdf. Accessed 3 Sep 2019.

[62] The King’s Fund (2015). How serious are the pressures in social care? Webpage. https://www.kingsfund.org.uk/projects/verdict/how-serious-are-pressures-social-care. Accessed 3 Sep 2019.

[63] The Mental Health Taskforce (2016). The Five Year Forward View for Mental Health. Resource document. https://www.england.nhs.uk/wp-content/uploads/2016/02/Mental-Health-Taskforce-FYFV-final.pdf. Accessed 3 Sep 2019.

[64] The King’s Fund (2018). Spending on public health. Webpage. https://www.kingsfund.org.uk/projects/nhs-in-a-nutshell/spending-public-health. Accessed 3 Sep 2019.

[65] British Medical Association (2019). Public health funding in England. Webpage. https://www.bma.org.uk/collective-voice/policy-and-research/public-and-population-health/public-health-budgets. Accessed 3 Sep 2019.

[66] MacIntyre A (1981). After virtue.

